# Medicare Advantage Enrollment and Disenrollment Among Persons With Alzheimer Disease and Related Dementias

**DOI:** 10.1001/jamahealthforum.2023.3080

**Published:** 2023-09-15

**Authors:** Hannah O. James, Amal N. Trivedi, David J. Meyers

**Affiliations:** 1 Department of Health Services, Policy, and Practice, Brown University School of Public Health, Providence, Rhode Island; 2 Providence Veterans Affairs Medical Center, Providence, Rhode Island

## Abstract

**Importance:**

Large enrollment growth has been observed in the Medicare Advantage program, but less is known about enrollment patterns among persons with Alzheimer disease and related dementias (ADRD).

**Objective:**

To evaluate patterns in Medicare Advantage enrollment and disenrollment among beneficiaries with or without ADRD.

**Design, Setting, and Participants:**

This cross-sectional study used 6 national data sources between January 1, 2011, and December 31, 2018. Analyses were performed between June 2021 and August 2022. The cohort comprised US Medicare beneficiaries with acute or postacute care utilization between 2013 and 2018.

**Exposure:**

ADRD diagnosis from an acute or postacute care encounter Medicare data source.

**Main Outcomes and Measures:**

Enrollment in Medicare Advantage, disenrollment from Medicare Advantage to traditional Medicare, and contract exit (leaving a Medicare Advantage contract for traditional Medicare or a different Medicare Advantage contract).

**Results:**

The 32 796 872 Medicare beneficiaries in the cohort had a mean (SD) age of 74.0 (12.5) years and included 18 228 513 females (55.6%). Enrollment in Medicare Advantage among beneficiaries with ADRD increased from 24.7% (95% CI, 24.7%-24.8%) in 2013 to 33.0% (95% CI, 32.9%-33.1%) in 2018, an absolute increase of 8.3 percentage points and a 33.4% relative increase after adjusting for demographic characteristics, comorbid conditions, and utilization and including county fixed effects. Among beneficiaries without ADRD, enrollment in Medicare Advantage increased by 8.2 percentage points from 27.6% (95% CI, 27.6%-27.6%) in 2013 to 35.8% (95% CI, 35.8%-35.8%) in 2018, a 29.7% relative increase over the study period. Beneficiaries with ADRD were 1.4 times as likely to disenroll from their Medicare Advantage contract to traditional Medicare (4.4% vs 3.2% in 2017-2018; *P* < .001) in adjusted analyses. Regardless of ADRD status, beneficiaries had similar rates of switching to a new Medicare Advantage contract. Differences in contract exit rates were associated with higher rates of disenrollment from Medicare Advantage to traditional Medicare among beneficiaries with ADRD vs those without ADRD (16.3% [95% CI, 16.2%-16.3%] vs 15.1% [95% CI, 15.1%-15.1%]). Beneficiaries with ADRD and dual eligibility for Medicaid enrollment had higher rates of contract exit than those without dual eligibility (19.7% [95% CI, 19.6%-19.7%] vs 14.9% [95% CI, 14.8%-14.9%]), and these differences were even greater than those among beneficiaries without ADRD and with and without dual-eligibility status, respectively (18.3% [95% CI, 18.2%-18.3%] vs 13.8% [95% CI, 13.7%-13.8%]).

**Conclusions and Relevance:**

In this cross-sectional study of the Medicare population with acute and postacute care use, beneficiaries with ADRD had increasing enrollment in the Medicare Advantage program, proportional to the growth in overall enrollment, but their disenrollment from Medicare Advantage in the following year remained higher compared with beneficiaries without ADRD. The findings highlight the need to understand the factors associated with higher disenrollment rates and determine whether such rates reflect access or quality challenges for beneficiaries with ADRD.

## Key Points

**Question:**

For people with Alzheimer disease and related dementias (ADRD), how have Medicare Advantage enrollment and disenrollment changed over time?

**Findings:**

In this repeated cross-sectional study of 32 796 872 unique Medicare beneficiaries with postacute or acute care utilization, Medicare Advantage enrollment increased from 2013 to 2018 among beneficiaries with ADRD, which was proportional to the overall enrollment growth among beneficiaries without ADRD. Despite this growth, beneficiaries with ADRD were less likely to remain enrolled in Medicare Advantage plans.

**Meaning:**

Findings of this study highlight the urgency to understand the factors associated with higher disenrollment rates and determine whether higher disenrollment rates reflect access or quality challenges for beneficiaries with ADRD.

## Teaser

This cross-sectional study assesses enrollment and disenrollment patterns for Medicare Advantage plans from 2013 to 2018 among beneficiaries with complex health conditions, including Alzheimer disease and related dementias.

## Introduction

More than 6 million individuals 65 years or older in the US are living with Alzheimer disease and related dementias (ADRD),^1^ for whom total payments for health care in 2021 were estimated at $355 billion.^2,3^ Persons with ADRD are more likely to be hospitalized, experience longer inpatient stays, and incur higher expenditures for their care compared with persons without ADRD.^4,5,6,7^ Annual mean per capita spending for beneficiaries with ADRD is estimated to be more than 3 times the mean costs for beneficiaries without ADRD, with out-of-pocket costs that are at least twice as high.^6,8,9^ Additionally, persons with ADRD experience more potentially avoidable hospitalizations and are likely to benefit from care management programs that are designed to prevent avoidable admissions.^10^

The Medicare Advantage program may be one avenue to improve the efficiency of health spending for persons with ADRD. Medicare Advantage plans receive capitated payments to manage care for their enrollees and therefore have financial incentives to improve the value of care delivery. Unlike traditional Medicare, Medicare Advantage plans must provide an annual patient out-of-pocket cost limit. They also frequently offer additional plan benefits (eg, nonemergency transportation) for services that are not typically covered by Medicare but may be valued by persons with ADRD and their caregivers.^11,12^ Moreover, enrollment in Medicare Advantage plans may be incentivized to encourage participation from healthier beneficiaries, on average, and restrict care that will not diminish overall quality.^13^ Prior research has identified that people with complex health care needs are disproportionately more likely to disenroll from Medicare Advantage in favor of traditional Medicare.^14,15^ Higher rates of voluntary disenrollment can be indicative of poor beneficiary experience and are important to evaluate as a signal of program success in meeting the needs of beneficiaries enrolled in the Medicare Advantage program.

Enrollment in Medicare Advantage is projected to surpass traditional Medicare enrollment nationally in 2023.^16^ Despite this tremendous growth, little is known about enrollment and disenrollment patterns over time among beneficiaries with complex health conditions, particularly ADRD. Prior studies of disenrollment evaluated data from a limited number of Medicare Advantage payers^17^ or a single year.^18^ National estimates characterizing Medicare Advantage enrollment among beneficiaries with ADRD are unavailable.

Differences in disenrollment by health status can indicate that a plan is not equipped to meet the needs of patients living with a particular condition. To the extent that differences in disenrollment are observed by health status, there may be cause for concern that Medicare Advantage plans are not serving the needs of the entire Medicare population. In this study, we aimed to evaluate patterns in Medicare Advantage enrollment and disenrollment among beneficiaries with or without ADRD between 2013 and 2018.

## Methods 

The Brown University Institutional Review Board approved the cross-sectional study and waived the informed consent requirement owing to the study’s inability to contact enrollees with deidentified claims data. We followed the Strengthening the Reporting of Observational Studies in Epidemiology (STROBE) reporting guideline.

### Data Sources and Cohort Construction

We used 6 sources of individual-level national data for Medicare Advantage and traditional Medicare beneficiaries from January 1, 2011, to December 31, 2018. The Medicare Provider Analysis and Review file provided information on inpatient stays in acute care hospitals. The Minimum Data Set provided information on nursing home care for all patients who were admitted to nursing homes. The Outcome and Assessment Information Set provided home health care utilization data for all beneficiaries. The Inpatient Rehabilitation Facility Patient Assessment Instrument data set provided information on inpatient rehabilitation facility utilization data for all beneficiaries. The Medicare Beneficiary Summary File (MBSF) provided demographic characteristics and monthly enrollment in traditional Medicare, Medicare Advantage, and Medicaid based on dual-eligibility status. The Healthcare Effectiveness Data and Information Set was used to identify the specific Medicare Advantage contract a beneficiary had enrolled in before this information was reported in the MBSF beginning in 2016.

Using national data from 2011 to 2018, we identified all Medicare beneficiaries with at least 3 years of Medicare enrollment and any hospital, postacute, or home health care utilization within a 3-year window. These data sets are collected for both Medicare Advantage and traditional Medicare enrollees and enable the study of a representative population of beneficiaries across both programs.^19^ Each of these data sources includes diagnosis codes that reflect beneficiary health status and can be used to identify groups of beneficiaries based on the presence of a health condition. In this study, we focused on persons with an ADRD diagnosis present in any of the utilization data sets.

To identify the study population of persons with ADRD, we required the presence of an *International Classification of Diseases, Ninth Revision* or *International Statistical Classification of Diseases and Related Health Problems, Tenth Revision* diagnostic code for ADRD, and we used the Chronic Conditions Data Warehouse definition for ADRD or senile dementia^20^ for any inpatient (Medicare Provider Analysis and Review file), nursing home (Minimum Data Set), home health (Outcome and Assessment Information Set), or inpatient rehabilitation (Inpatient Rehabilitation Facility Patient Assessment Instrument) encounter (eMethods in Supplement 1). We used a 3-year look-back period, consistent with the procedure followed for Chronic Conditions Data Warehouse definitions, to better capture ADRD diagnoses that might not be evident within a single year. At least 1 ADRD diagnosis from 1 of the acute or postacute care data sources during the look-back period was sufficient for inclusion in the ADRD study group. Alzheimer disease and related dementias are chronic conditions without available curative treatments; thus, beneficiaries with a documented ADRD diagnosis remained in the ADRD study population until their death. Those without any ADRD diagnoses recorded in a 3-year look-back period composed the study group of beneficiaries without ADRD. We constructed annual cross-sectional cohorts of beneficiaries with and without ADRD for comparison for each study year between 2013 and 2018 (eFigure 1 in Supplement 1).

### Measures

The 2 primary study outcomes were Medicare Advantage enrollment and disenrollment. We defined enrollment in Medicare Advantage as enrollment by December in each study year. We also characterized enrollment by other definitions, such as at least 1 month of enrollment, enrollment for most months of the year, and full enrollment, and the findings remained consistent. We defined disenrollment from Medicare Advantage as enrollment in December of the baseline year and enrollment in traditional Medicare in December of the following year.

For disenrollment analyses, the eligible population for study included enrollees in a Medicare Advantage contract in December of a baseline year who remained alive in December of the subsequent year, who did not move residence (as reflected by a change in the county indicator from the MBSF across years; <4% per year), and whose baseline year contract was not terminated administratively (<0.05% per year). For each study year, we assessed whether enrollees in Medicare Advantage in December of a baseline year subsequently (1) disenrolled from Medicare Advantage to enroll in traditional Medicare, (2) stayed in Medicare Advantage but enrolled in a different Medicare Advantage contract, or (3) remained in the same Medicare Advantage contract in January of the following year.

A secondary outcome was contract exit, defined as either exiting an existing Medicare Advantage contract for a different Medicare Advantage contract or for traditional Medicare in the following year. Covariates derived from the MBSF included age, sex, race and ethnicity (self-reported as Asian or Pacific Islander, Black, Hispanic, non-Hispanic White, and other [including American Indian or Alaska Native, other, or unknown]), dual-eligibility enrollment status, and reason for entitlement. Race and ethnicity data were collected and included in analysis because enrollment in Medicare Advantage has varied according to racial and ethnic identity. Other covariates were derived from the utilization data sources to reflect additional indicators of health status (indicators for diabetes, heart failure, chronic obstructive pulmonary disease, acute myocardial infarction, and schizophrenia and other serious mental illness) and binary indicators of inpatient, nursing home, and home health utilization in each study year.

### Statistical Analysis

After defining the cohort, we descriptively compared the characteristics of beneficiaries enrolled in Medicare Advantage vs traditional Medicare by ADRD status. We then evaluated enrollment in Medicare Advantage as a proportion of the total eligible population of beneficiaries with or without ADRD who were enrolled in a Medicare Advantage contract in December of each study year (2013 to 2018). We used Medicare Advantage enrollment indicators from the MBSF, adjusting for potential differences in age, sex, race and ethnicity, dual-eligibility enrollment status, additional health conditions, and acute and postacute care utilization in a multivariable linear regression model with county fixed effects to account for the differential opportunity to enroll in Medicare Advantage by county based on plan offerings. Beneficiary-level demographic characteristics, health status indicators, and utilization indicators were included to adjust for differences based on ADRD status. We then plotted patterns in adjusted enrollment in Medicare Advantage over time for beneficiaries with or without ADRD. We also estimated overall growth in enrollment using a pooled multivariable linear regression model with year fixed effects.

We estimated adjusted disenrollment rates using a multinomial logistic regression model, including covariates for age, sex, race and ethnicity, dual-eligibility enrollment status, health status, and utilization indicators for each study period (2013-2014, 2014-2015, 2015-2016, 2016-2017, and 2017-2018), among beneficiaries enrolled in Medicare Advantage in the baseline year who remained alive in the following enrollment year. We calculated the adjusted overall disenrollment rate for the entire study period separately in a pooled multinomial logistic regression model that also included indicators for each study period. In sensitivity analyses, we evaluated a binary outcome for contract exit (stayed vs exited the Medicare Advantage contract) by dual-eligibility enrollment status, sex, and race and ethnicity while controlling for all other demographic variables, such as age and other health status and utilization indicators. In further sensitivity analyses, we assessed the data sources of ADRD diagnoses (eFigure 2 in Supplement 1) and tested alternative look-back periods for an ADRD diagnosis (eFigure 3 in Supplement 1).

For all analyses, we used robust SEs in each statistical model. We calculated margins to compute the adjusted means from both the multivariable linear regression and the multinomial logistic regression models. All *P* values were calculated from 2-sided significance tests using an α = .05 to indicate statistical significance. All analyses were conducted between June 2021 and August 2022 using Stata, version 16.1 (StataCorp LLC).

## Results

The study included 32 796 872 unique Medicare beneficiaries with acute or postacute care utilization between 2013 and 2018; of these beneficiaries, 18 228 513 were females (55.6%) and 14 568 359 were males (44.4%) with a mean (SD) age of 74.0 (12.5) years. For each study year, there were approximately 16 529 641 beneficiaries meeting the overall inclusion criteria, and a mean (SD) 14.5% (0.1%) of the eligible study population in each year had evidence of ADRD. Beneficiaries with ADRD vs without ADRD were more likely to be older (mean [SD] age, 82.0 [9.8] vs 72.7 [12.0] years in 2018), female (63.7% vs 55.2% in 2018), and enrolled in Medicaid (41.9% vs 25.1% in 2018). Beneficiaries with ADRD vs without ADRD were more likely to have other comorbid conditions (eg, 28.8% vs 19.3% also had a heart failure diagnosis in 2018). Beneficiaries with ADRD vs without ADRD had a lower prevalence of inpatient utilization over a 3-year period from 2016 to 2018 (82.2% vs 90.7%) but were much more likely to have nursing home (62.7% vs 19.0%) or home health (61.3% vs 39.0%) utilization (Table 1).

**Table 1.** Sample Characteristics of Medicare Enrollees by ADRD Status for 2013 and 2018

Characteristic	Medicare enrollees, No. (%)			
	2013		2018	
	Without ADRD	With ADRD	Without ADRD	With ADRD
All	13 663 315	2 336 906	14 561 088	2 465 204
Age, mean (SD), y	72.5 (12.6)	82.7 (9.2)	72.7 (12.0)	82.0 (9.8)
Sex				
Female	7 659 866 (56.1)	1 557 459 (66.6)	8 040 089 (55.2)	1 571 367 (63.7)
Male	6 003 449 (43.9)	779 447 (33.4)	6 520 999 (44.8)	893 837 (36.3)
Race and ethnicity^a^				
Asian or Pacific Islander	265 149 (1.9)	47 795 (2.0)	315 677 (2.2)	60 405 (2.5)
Black	1 531 343 (11.2)	284 654 (12.2)	1 654 015 (11.4)	302 406 (12.3)
Hispanic	1 029 026 (7.5)	169 445 (7.3)	1 192 872 (8.2)	198 430 (8.0)
Non-Hispanic White	10 637 303 (77.9)	1 811 594 (77.5)	11 066 487 (76.0)	1 868 172 (75.8)
Other^b^	200 494 (1.5)	23 418 (1.0)	332 037 (2.3)	35 791 (1.5)
Medicaid dual-eligibility enrollment	3 458 496 (25.3)	1 020 085 (43.7)	3 654 372 (25.1)	1 032 975 (41.9)
Reason for entitlement				
OASI	10 746 339 (78.7)	2 225 276 (95.2)	12 046 051 (82.7)	2 349 844 (95.3)
DIB	2 772 370 (20.3)	105 583 (4.5)	2 456 481 (16.9)	113 131 (4.6)
ESKD	60 304 (0.4)	3541 (0.2)	37 326 (0.3)	1167 (<0.01)
Both DIB and ESKD	84 132 (0.6)	2485 (0.1)	21 230 (0.1)	1062 (<0.01)
Comorbid conditions				
Diabetes	4 384 256 (32.1)	746 099 (31.9)	4 815 126 (33.1)	838 852 (34.0)
Heart failure	2 435 001 (17.8)	635 174 (27.2)	2 811 891 (19.3)	709 745 (28.8)
COPD	2 781 403 (20.4)	544 676 (23.3)	3 126 749 (21.5)	608 291 (24.7)
SMI	510 459 (3.7)	295 015 (12.6)	430 504 (3.0)	203 022 (8.2)
AMI	712 888 (5.2)	128 399 (5.5)	924 721 (6.4)	181 056 (7.3)
3-y Utilization^c^				
Inpatient	12 393 758 (90.7)	1 890 219 (80.9)	13 211 554 (90.7)	2 025 597 (82.2)
Nursing home	2 587 178 (18.9)	1 484 846 (63.5)	2 770 693 (19.0)	1 545 875 (62.7)
Home health	5 519 971 (40.4)	1 405 898 (60.2)	5 679 137 (39.0)	1 511 089 (61.3)

Abbreviations: ADRD, Alzeheimer disease and related dementias; AMI, acute myocardial infarction; COPD, chronic obstructive pulmonary disease; DIB, disability insurance benefits; ESKD, end-stage kidney disease; OASI, Old Age and Survivors Insurance; SMI, schizophrenia and other serious mental illness.

^a^ Race and ethnicity were self-reported by beneficiaries and obtained from the Medicare Beneficiary Summary File.

^b^ Other included American Indian or Alaska Native, other, or unknown.

^c^ Utilization reflects beneficiaries with any utilization during the study year and the prior 2 years (eg, 2013 study population included those with utilization between 2011 and 2013) from the Medicare Provider Analysis and Review, Outcome and Assessment Information Set, Inpatient Rehabilitation Facility Patient Assessment Instrument, and Minimum Data Set. Beneficiaries commonly have multiple utilization events during a period across different data sources.

**Figure 1.**
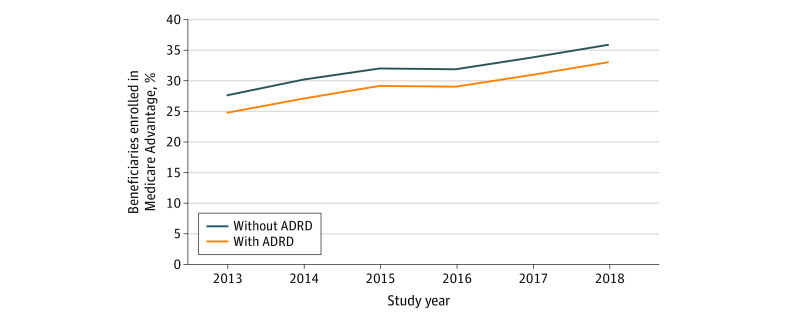
Estimates of Adjusted Medicare Advantage Enrollment Patterns by Alzheimer Disease and Related Dementias (ADRD) Status From 2013 to 2018 Estimates were calculated using margins following estimation of a multivariable linear regression model, including covariates for age; sex; race and ethnicity; dual-eligibility enrollment status; indicators for diabetes, heart failure, chronic obstructive pulmonary disease, acute myocardial infarction, and schizophrenia and other serious mental illness; and yearly indicators for inpatient, nursing home, and home health utilization.

Among beneficiaries with ADRD, unadjusted enrollment in Medicare Advantage increased by 9.5 percentage points from 22.6% in 2013 to 32.1% in 2018. Unadjusted enrollment in Medicare Advantage among those without ADRD increased by 8.0 percentage points from 28.0% to 36.0% over the study period. Among those with ADRD, adjusted enrollment in Medicare Advantage increased from 24.7% (95% CI, 24.7%-24.8%) in 2013 to 33.0% (95% CI, 32.9%-33.1%) in 2018, an absolute increase of 8.3 percentage points and a 33.4% relative increase over the study period. Adjusted Medicare Advantage enrollment among those without ADRD increased from 27.6% (95% CI, 27.6%-27.6%) in 2013 to 35.8% (95% CI, 35.8%-35.8%) in 2018, an absolute increase of 8.2 percentage points and a 29.7% relative increase (Figure 1; eTable 1 in Supplement 1).

All racial and ethnic groups experienced increases in Medicare Advantage enrollment, with enrollment among beneficiaries with ADRD slightly lagging compared with that among beneficiaries without ADRD. Enrollment rates varied considerably by race and ethnicity among beneficiaries with ADRD, ranging from 48.9% (95% CI, 48.7%-49.1%) among Hispanic beneficiaries to 26.0% (95% CI, 25.4%-26.5%) among beneficiaries who reported other race and ethnicity in 2018 (Table 2).

**Table 2.** Estimated Medicare Advantage Enrollment Stratified by Race and Ethnicity and Dual-Eligibility Enrollment Status by Alzheimer Disease and Related Dementias (ADRD) Status for 2013 and 2018

Characteristic	Estimated enrollment, % (95% CI)^a^			
	2013		2018	
	Without ADRD	With ADRD	Without ADRD	With ADRD
Race and ethnicity^b^				
Asian or Pacific Islander	29.5 (29.3-29.6)	27.2 (26.8-27.6)	37.7 (37.5-37.8)	35.4 (35.0-35.8)
Black	30.5 (30.5-30.6)	26.1 (25.9-26.2)	43.3 (43.2-43.3)	38.4 (38.2-38.6)
Hispanic	40.6 (40.5-40.6)	36.8 (36.5-37.0)	52.9 (52.8-53.0)	48.9 (48.7-49.1)
Non-Hispanic White	26.0 (26.0-26.0)	23.3 (23.2-23.3)	33.0 (33.0-33.0)	30.5 (30.4-30.6)
Other^c^	22.4 (22.2-22.6)	20.3 (19.8-20.9)	28.3 (28.1-28.4)	26.0 (25.4-26.5)
Dual-eligibility enrollment status				
Non–dually eligible	28.6 (28.6-28.7)	26.3 (26.2-26.4)	35.1 (35.0-35.1)	33.2 (33.1-33.3)
Dually eligible	25.1 (25.0-25.1)	20.9 (20.8-21.0)	37.8 (37.8-37.9)	33.2 (33.1-33.3)

^a^ Estimates were calculated using margins following estimation of a multivariable linear regression model, including covariates for age; sex; indicators for diabetes, heart failure, chronic obstructive pulmonary disease, acute myocardial infarction, and schizophrenia and other serious mental illness; and yearly indicators for inpatient, nursing home, and home health utilization.

^b^ Race and ethnicity data relied on the Research Triangle Institute race variable in the Medicare Beneficiary Summary File, which enhances the beneficiary race code variable to more accurately identify beneficiaries as Hispanic or Asian based on surname and other geographic indicators.

^c^ Other included American Indian or Alaska Native, other, or unknown.

**Figure 2.**
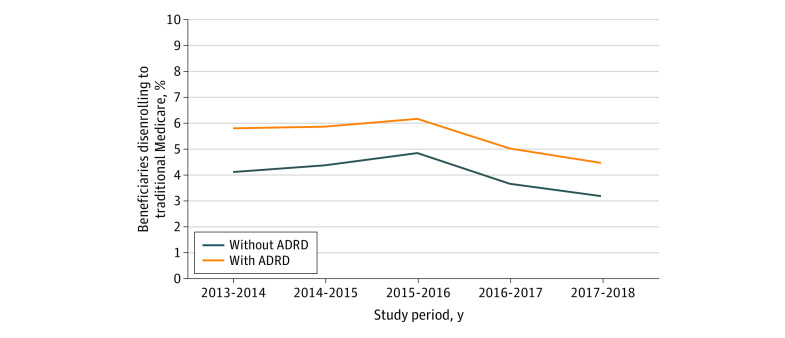
Estimates of Adjusted Percentage of Medicare Advantage Enrollees Disenrolling to Traditional Medicare by Alzheimer Disease and Related Dementias (ADRD) Status From 2013 to 2018 Estimates were calculated using margins following estimation of a multivariable linear regression model, including covariates for age; sex; race and ethnicity; dual-eligibility enrollment status; indicators for diabetes, heart failure, chronic obstructive pulmonary disease, acute myocardial infarction, and schizophrenia and other serious mental illness; and yearly indicators for inpatient, nursing home, and home health utilization.

Over the entire study period, 6.3% (95% CI, 6.2%-6.3%) of beneficiaries with ADRD who were enrolled in Medicare Advantage disenrolled to traditional Medicare compared with 3.9% (95% CI, 3.9%-3.9%) of beneficiaries without ADRD in unadjusted analyses. After adjusting for demographic characteristics, health status, and utilization, the difference in disenrollment rates remained significantly higher among beneficiaries with ADRD. In adjusted analyses, 5.0% (95% CI, 4.9%-5.0%) of beneficiaries with ADRD disenrolled to traditional Medicare compared with 4.0% (95% CI, 4.0%-4.0%) of beneficiaries without ADRD. Between 2013 and 2018, disenrollment rates ranged from 4.4% (95% CI, 4.4%-4.5%) to 6.1% (95% CI, 6.1%-6.2%) for beneficiaries with ADRD and from 3.2% (95% CI, 3.2%-3.2%) to 4.8% (95% CI, 4.8%-4.8%) for beneficiaries without ADRD. From 2017 to 2018, beneficiaries with ADRD who were enrolled in Medicare Advantage were 1.4 times as likely to disenroll to traditional Medicare compared with beneficiaries without ADRD (4.4% vs 3.2%; *P* < .001) (Figure 2).

Beneficiaries with ADRD had higher rates of disenrolling to traditional Medicare across all racial and ethnic categories and dual-eligibility enrollment status. Disenrollment rates among beneficiaries with ADRD ranged from 4.9% (95% CI, 4.8%-4.9%) among non-Hispanic White beneficiaries to 6.2% (95% CI, 6.1%-6.2%) among Asian or Pacific Islander beneficiaries. Disenrollment rates were twice as high among beneficiaries with ADRD who were dually eligible for enrollment in Medicaid vs those without dual eligibility (7.8% [95% CI, 7.7%-7.8%] vs 3.7% [95% CI, 3.7%-3.7%]) (eTable 2 in Supplement 1). Beneficiaries with ADRD had 24.0% higher rates of disenrollment across all racial and ethnic groups.

**Figure 3.**
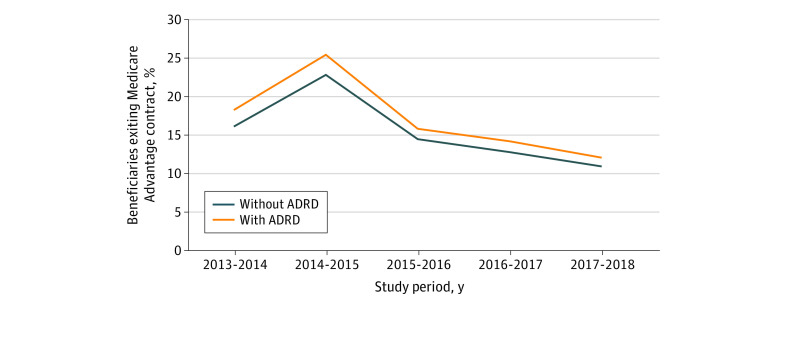
Estimates of Adjusted Percentage of Medicare Advantage Enrollees Exiting Medicare Advantage Contracts by Alzheimer Disease and Related Dementias (ADRD) Status From 2013 to 2018 Estimates were calculated using margins following estimation of a multivariable logistic regression with a binary outcome: remain or exit contract, where exit was a composite indicator for disenrolling to traditional Medicare or changing to a new Medicare Advantage contract.

Beneficiaries had similar rates of changing their contract but staying in Medicare Advantage (11.1%) regardless of ADRD status. Given the similarities in the proportion of beneficiaries switching to a new contract within the Medicare Advantage program, the difference in the contract exit outcome by ADRD status was primarily associated with differences in disenrollment to traditional Medicare. Over the study period, 16.3% (95% CI, 16.2%-16.3%) of beneficiaries with ADRD exited their Medicare Advantage contract compared with 15.1% (95% CI, 15.1%-15.1%) among beneficiaries without ADRD (eTable 3 in Supplement 1). We observed variation in the contract exit rate by beneficiary race and ethnicity. Hispanic beneficiaries with ADRD had the highest contract exit rate at 18.2% (95% CI, 18.1%-18.2%), which was 1.2 times greater than the rate of contract exit among beneficiaries with ADRD with the lowest contract exit rate (eTable 4 in Supplement 1). For beneficiaries with ADRD and dual-eligibility Medicaid enrollment, 19.7% (95% CI, 19.6%-19.7%) exited their contract compared with 14.9% (95% CI, 14.8%-14.9%) of beneficiaries with ADRD without dual-eligibility enrollment. Contract exit rates appeared to have declined and stabilized during the 2017-to-2018 period, with 12.0% (95% CI, 11.9%-12.1%) of beneficiaries with ADRD exiting their Medicare Advantage contract compared with 10.9% (95% CI, 10.9%-10.9%) of beneficiaries without ADRD (Figure 3).

## Discussion

This study presented 3 key findings. First, from 2013 to 2018, there was substantial growth in enrollment in Medicare Advantage among beneficiaries with ADRD, which was similar in magnitude to the increase in enrollment among beneficiaries without ADRD. Second, beneficiaries with ADRD were more likely to leave their Medicare Advantage contract compared with beneficiaries without ADRD. Beneficiaries with and without ADRD had similar rates of switching contracts within the Medicare Advantage program, but during the entire study period, beneficiaries with ADRD were more likely to disenroll to traditional Medicare. Third, there were differences in contract exit rates associated with a beneficiary’s race and ethnicity and dual-eligibility enrollment status for Medicaid. Hispanic beneficiaries with ADRD had the highest rate of exiting their Medicare Advantage contract compared with their counterparts in other racial and ethnic groups. This finding could be associated with the greater likelihood of lower-income populations to enroll in lower-performing Medicare Advantage plans^21,22,23^ or plans with more restrictive benefits or networks, which are often less expensive for beneficiaries to purchase. The findings for individuals with dual-eligibility enrollment in Medicaid are aligned with results of prior work showing higher rates of disenrollment for this population, particularly among beneficiaries with acute or postacute care utilization.^14^

This study builds on prior work by evaluating a longer timespan to better understand the changes over time in Medicare Advantage enrollment and disenrollment for beneficiaries with ADRD and potential differences based on dual-eligibility enrollment status and race and ethnicity. A study^17^ using data from 3 Medicare Advantage payers found higher all-cause disenrollment (switching plans, disenrolling to traditional Medicare, and death) among beneficiaries with ADRD but could not disaggregate the reasons for disenrollment due to data limitations. Another study^18^ found higher disenrollment rates among beneficiaries with ADRD and higher rates of disenrollment among beneficiaries with poorer cognitive functioning but was only able to evaluate 1 year of data.

Despite similar patterns in increasing Medicare Advantage enrollment, beneficiaries with ADRD enrolled in a Medicare Advantage contract had consistently higher rates of contract exit and switch to traditional Medicare compared with beneficiaries without ADRD. This finding suggests that for a proportion of beneficiaries with ADRD, their Medicare Advantage contract may not meet their needs. This finding was contrary to that of a prior study that used a smaller sample of Medicare beneficiaries and reported that beneficiaries with ADRD who were enrolled in Medicare Advantage had lower rates of health care utilization that did not adversely affect care satisfaction and health status, but the study did not evaluate disenrollment.^24^ Beneficiaries with ADRD and their caregivers may face challenges in navigating the narrow networks common in the Medicare Advantage program as well as more complicated plan designs that may require prior authorization and more administrative barriers to access care.^25^ Given the heterogeneity of plan design and the volume of plans available in some US counties, it is possible that beneficiaries with ADRD and their caregivers may face substantial challenges in identifying a suitable Medicare Advantage plan; beneficiaries with ADRD who lack sufficient social support may be further disadvantaged. The findings of this study are consistent with those of prior work demonstrating that persons with complex health care needs have higher rates of disenrolling from Medicare Advantage in favor of traditional Medicare.^14,15^

### Limitations

This study has 3 main limitations. First, our ability to identify beneficiaries with ADRD in the Medicare program relied on beneficiaries’ use of hospital, nursing home, home health, or inpatient rehabilitation care. This means that the study population was likely to be sicker on average (regardless of ADRD status), and therefore the results may not be generalizable to all Medicare beneficiaries. Second, we lacked information on beneficiaries’ specific reasons for Medicare Advantage enrollment to confirm the potential mechanisms behind these enrollment patterns over time. Third, we lacked information on beneficiaries’ cohabitation or marital status, which may be an important factor in both enrollment and disenrollment decisions.

## Conclusions

In this repeated cross-sectional study, we identified a pattern of growth in Medicare Advantage enrollment among Medicare beneficiaries with ADRD that mirrors the increasing enrollment pattern among beneficiaries without ADRD. Across all study years, beneficiaries with ADRD were consistently more likely to disenroll from Medicare Advantage or leave their existing Medicare Advantage contract compared with persons without ADRD. These findings highlight the urgency to better understand the factors associated with higher disenrollment rates for beneficiaries with ADRD and to determine whether higher disenrollment rates reflect challenges with access to care or quality of care for this high-cost, high-need population. Policy responses that heighten the oversight of plans with higher rates of disenrollment among enrollees with chronic illness (in addition to overall measures of disenrollment) and increased oversight and enforcement of existing network adequacy rules are essential to ensure that the Medicare Advantage program is meeting the needs of all enrollees. Further research is needed to identify Medicare Advantage plans that can best support the dynamic needs of persons with ADRD and their caregivers.

## Notes

Supplement 1. **eFigure 1.** Study Population Flowchart, 2018 **eFigure 2.** Source of ADRD Identification by Assessment Data Source, 2011-2018 **eFigure 3.** Evaluation of One, Two, and Three-Year Lookback Periods for Identifying ADRD Population **eTable 1.** Linear Regression Estimating Adjusted Medicare Advantage Enrollment, 2013-2018 **eTable 2.** Adjusted Rate of Disenrollment to Traditional Medicare by Race/Ethnicity and Dual Medicaid Enrollment **eTable 3.** Logistic Regression Estimating Adjusted Contract Exit, 2013-2018 **eTable 4.** Adjusted Rate of Contract Exit by Race/Ethnicity and Dual Medicaid Enrollment **eMethods.** Chronic Conditions Warehouse Criteria for Alzheimer’s Disease and Related Disorders or Senile Dementia

Supplement 2.
Data Sharing Statement
