# Supplementary material for: Medicare Advantage Enrollment and Disenrollment Among Persons With Alzheimer Disease and Related Dementias
Source: JAMA Health Forum. 2023 Sep 15;4(9):e233080. doi: 10.1001/jamahealthforum.2023.3080 (PMC10504614; doi:10.1001/jamahealthforum.2023.3080)
Supplement: Supplement 1. — eFigure 1. Study Population Flowchart, 2018 eFigure 2. Source of ADRD Identification by Assessment Data Source, 2011-2018 eFigure 3. Evaluation of One, Two, and Three-Year Lookback Periods for Identifying ADRD Population eTable 1. Linear Regression Estimating Adjusted Medicare Advantage Enrollment, 2013-2018 eTable 2. Adjusted Rate of Disenrollment to Traditional Medicare by Race/Ethnicity and Dual Medicaid Enrollment eTable 3. Logistic Regression Estimating Adjusted Contract Exit, 2013-2018 eTable 4. Adjusted Rate of Contract Exit by Race/Ethnicity and Dual Medicaid Enrollment eMethods. Chronic Conditions Warehouse Criteria for Alzheimer’s Disease and Related Disorders or Senile Dementia [file jamahealthforum-e233080-s001.pdf]

Supplementary Online Content

James HO, Trivedi AN, Meyers DJ. Medicare Advantage enrollment and disenrollment among persons with Alzheimer disease and related dementias. *JAMA Health Forum.* Published online September 15, 2023. doi:10.1001/jamahealthforum.2023.3080

eFigure 1. Study Population Flowchart, 2018

eFigure 2. Source of ADRD Identification by Assessment Data Source, 2011-2018

eFigure 3. Evaluation of One, Two, and Three-Year Lookback Periods for Identifying ADRD Population

eTable 1. Linear Regression Estimating Adjusted Medicare Advantage Enrollment, 2013-2018

eTable 2. Adjusted Rate of Disenrollment to Traditional Medicare by Race/Ethnicity and Dual Medicaid Enrollment

eTable 3. Logistic Regression Estimating Adjusted Contract Exit, 2013-2018

eTable 4. Adjusted Rate of Contract Exit by Race/Ethnicity and Dual Medicaid Enrollment

eMethods. Chronic Conditions Warehouse Criteria for Alzheimer's Disease and Related Disorders or Senile Dementia

This supplementary material has been provided by the authors to give readers additional information about their work.

© 2023 James HO et al. *JAMA Health Forum.*

eFigure 1. Study population flowchart, 2018

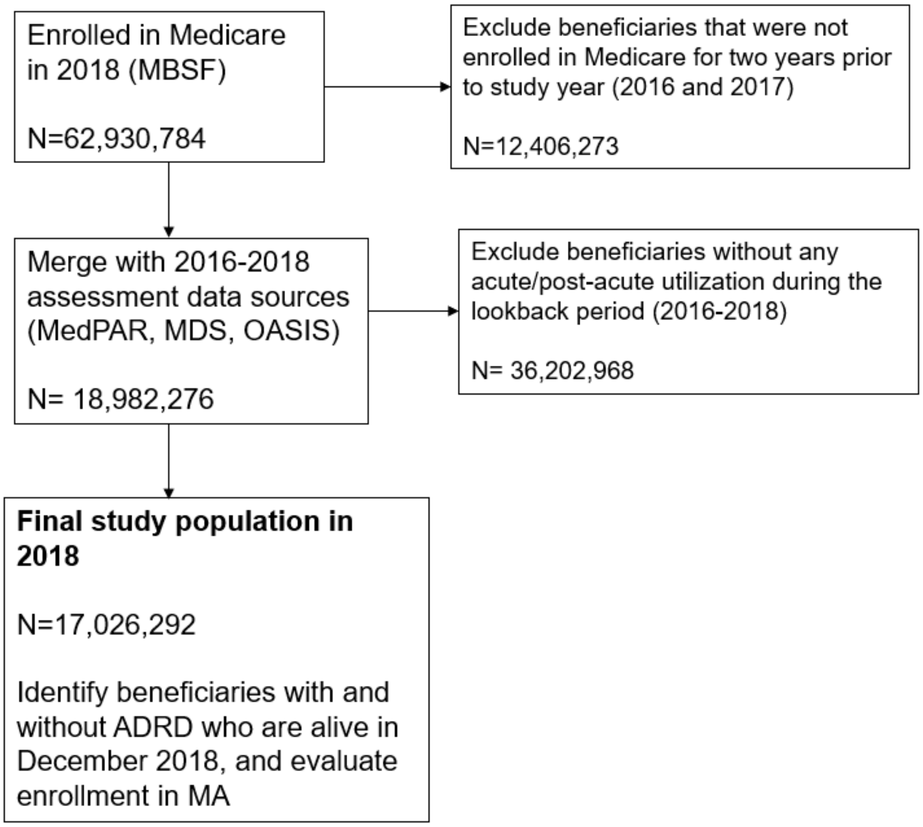

Notes: The flowchart includes values reflecting the 2018 study cohort only; the same process was followed for all other study cohorts included in the study period, but exact numeric values differ for each study period.

© 2023 James HO et al. *JAMA Health Forum.*

eFigure 2. Source of ADRD identification by assessment data source, 2011-2018

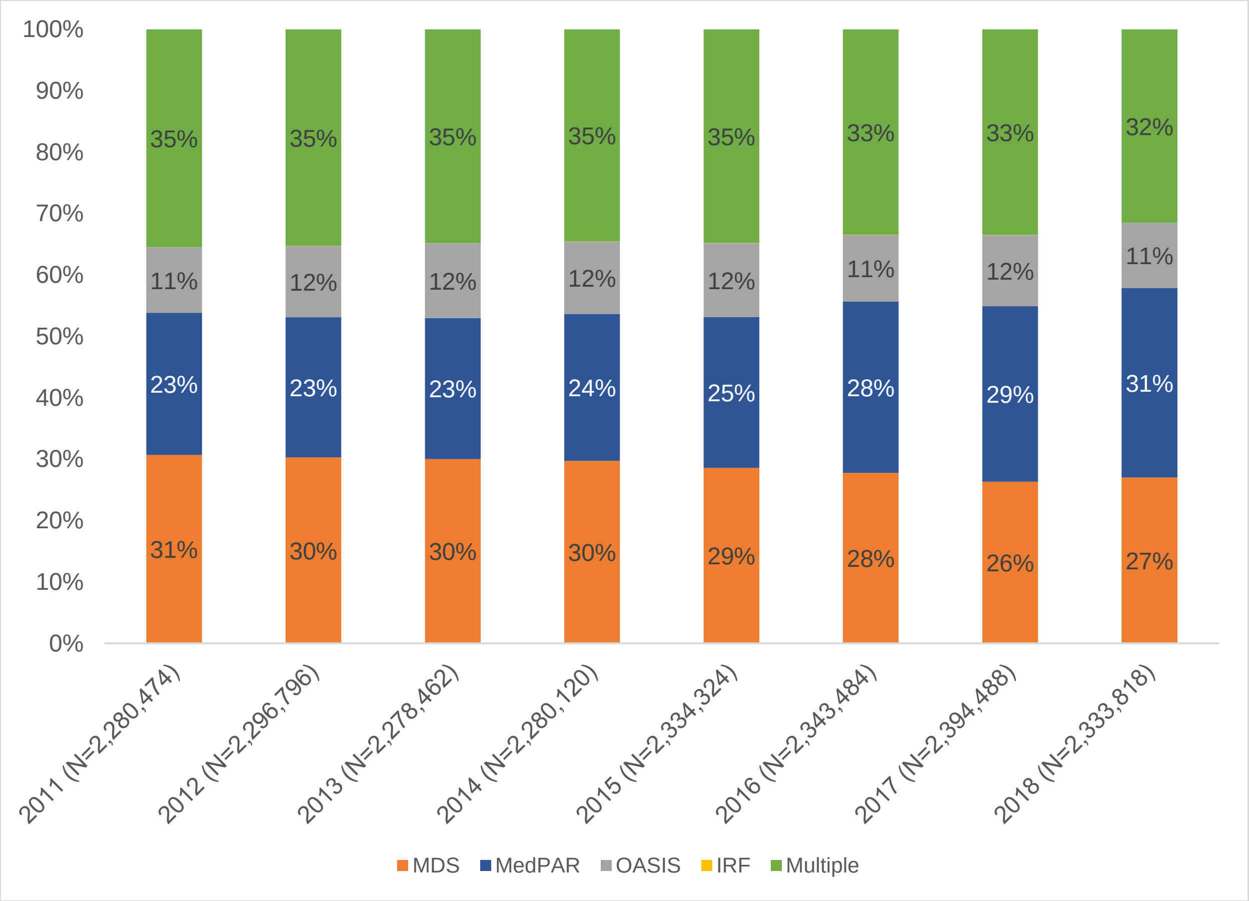

Notes: Single-year study of source of ADRD identification by utilization data source – for reference only to show stability in source of ADRD diagnoses; study population relies on 3-year lookback period.

© 2023 James HO et al. *JAMA Health Forum.*

eFigure 3. Evaluation of one, two, and three-year lookback periods for identifying ADRD population

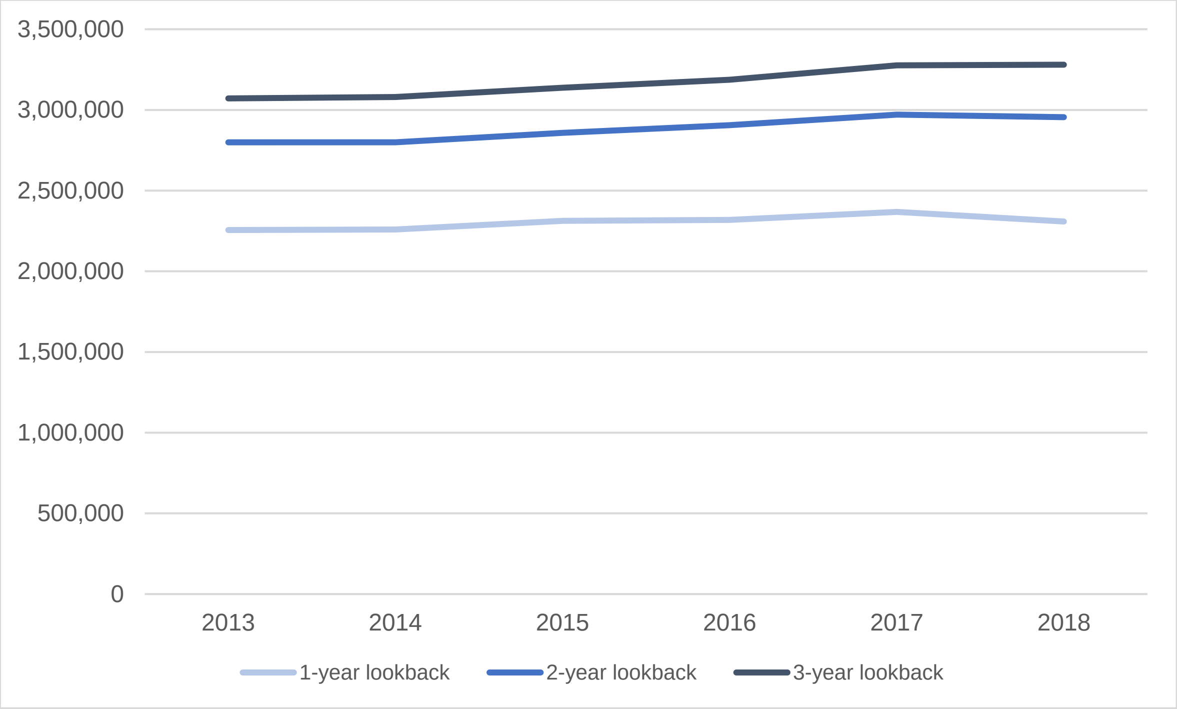

Notes: We evaluated different lookback periods to determine if this would have an impact on the identification of our study population. For each of these lookback definitions, the denominator was held constant (required to be enrolled in Medicare for 3 years, so for 2013: 2011-2013) and then varying the window (one, two, or 3 years) to capture ADRD diagnosis from assessment data sources. By using a three-year lookback period, we identify ~30% additional beneficiaries with an ADRD diagnosis each year.

© 2023 James HO et al. *JAMA Health Forum.*

eTable 1. Linear regression estimating adjusted Medicare Advantage enrollment, 2013-2018

	2013	2014	2015	2016	2017	2018
Age						
Under 65	Ref.					
65-75	0.0576***	0.0491***	0.0550***	0.0569***	0.0496***	0.0491***
	(0.000765)	(0.00469)	(0.00825)	(0.00745)	(0.00748)	(0.00758)
75-85	0.0579***	0.0522***	0.0605***	0.0663***	0.0641***	0.0663***
	(0.000818)	(0.00469)	(0.00825)	(0.00746)	(0.00748)	(0.00759)
85+	0.0320***	0.0248***	0.0332***	0.0398***	0.0402***	0.0413***
	(0.000847)	(0.00470)	(0.00826)	(0.00746)	(0.00748)	(0.00759)
Race/ethnicity						
White Non-Hispanic	Ref.					
Black	0.0737***	0.0782***	0.0828***	0.0883***	0.0957***	0.105***
	(0.000375)	(0.000384)	(0.000389)	(0.000385)	(0.000388)	(0.000392)
API	0.0338***	0.0494***	0.0610***	0.0389***	0.0362***	0.0353***
	(0.000821)	(0.000841)	(0.000845)	(0.000813)	(0.000805)	(0.000807)
Hispanic Non-White	0.158***	0.170***	0.178***	0.186***	0.189***	0.194***
	(0.000477)	(0.000479)	(0.000477)	(0.000467)	(0.000463)	(0.000461)
Other	-0.0290***	-0.0338***	-0.0349***	-0.0388***	-0.0433***	-0.0454***
	(0.000878)	(0.000862)	(0.000839)	(0.000792)	(0.000765)	(0.000750)
Female	0.0120***	0.0133***	0.0136***	0.0140***	0.0156***	0.0158***
	(0.000226)	(0.000230)	(0.000232)	(0.000230)	(0.000231)	(0.000234)
Dual enrollment	-0.0421***	-0.0212***	-0.00102***	-0.0130***	-0.00848***	0.00446***
	(0.000288)	(0.000294)	(0.000299)	(0.000297)	(0.000300)	(0.000305)
Reason for entitlement						
OASI	Ref.					
DIB	0.00638***	0.00188	0.00782	0.0159**	0.0146*	0.0210***
	(0.000747)	(0.00470)	(0.00826)	(0.00746)	(0.00748)	(0.00759)
ESRD	-0.202***	-0.246***	-0.241***	-0.247***	-0.265***	-0.282***
	(0.00122)	(0.00457)	(0.00817)	(0.00736)	(0.00737)	(0.00748)
Both DIB & ESRD	-0.171***	-0.199***	-0.193***	-0.152***	-0.158***	-0.164***
	(0.00123)	(0.00478)	(0.00837)	(0.00773)	(0.00784)	(0.00803)
ADRD	-0.0288***	-0.0308***	-0.0279***	-0.0286***	-0.0278***	-0.0281***
	(0.000352)	(0.000362)	(0.000368)	(0.000362)	(0.000364)	(0.000369)
Utilization						
Nursing home, Year of enrollment	0.00592***	0.00289***	0.00734***	0.00732***	0.00782***	0.00689***
	(0.000372)	(0.000377)	(0.000380)	(0.000378)	(0.000383)	(0.000393)
Nursing home, Y-1	-0.00582***	-0.00480***	-0.00390***	-0.00113***	-0.00456***	-0.00388***

© 2023 James HO et al. *JAMA Health Forum.*

	(0.000419)	(0.000424)	(0.000427)	(0.000421)	(0.000428)	(0.000435)
Nursing home, Y-2	-0.0113***	-0.0108***	-0.00677***	-0.0106***	-0.00855***	-0.0115***
	(0.000430)	(0.000437)	(0.000442)	(0.000436)	(0.000441)	(0.000449)
Inpatient, Year of enrollment	-0.0195***	-0.0191***	-0.0240***	-0.0259***	-0.0296***	-0.0379***
	(0.000248)	(0.000255)	(0.000258)	(0.000256)	(0.000258)	(0.000260)
Inpatient, Y-1	-0.0189***	-0.0166***	-0.0172***	-0.0198***	-0.0208***	-0.0239***
	(0.000246)	(0.000254)	(0.000258)	(0.000256)	(0.000258)	(0.000260)
Inpatient, Y-2	-0.0179***	-0.0169***	-0.0161***	-0.0139***	-0.0152***	-0.0161***
	(0.000246)	(0.000254)	(0.000260)	(0.000259)	(0.000261)	(0.000264)
Home health, Year of enrollment	-0.0323***	-0.0312***	-0.0322***	-0.0323***	-0.0279***	-0.0192***
	(0.000283)	(0.000288)	(0.000290)	(0.000287)	(0.000292)	(0.000316)
Home health, Y-1	-0.0194***	-0.0229***	-0.0206***	-0.0256***	-0.0234***	-0.0204***
	(0.000309)	(0.000312)	(0.000316)	(0.000311)	(0.000315)	(0.000324)
Home health, Y-2	-0.0272***	-0.0243***	-0.0244***	-0.0288***	-0.0292***	-0.0277***
	(0.000324)	(0.000319)	(0.000322)	(0.000319)	(0.000323)	(0.000328)
Other health conditions						
Heart failure	0.00313***	0.00155***	0.00241***	0.00208***	0.00313***	0.00481***
	(0.000304)	(0.000310)	(0.000314)	(0.000310)	(0.000311)	(0.000312)
AMI	0.0180***	0.0169***	0.0178***	0.0196***	0.0198***	0.0211***
	(0.000505)	(0.000511)	(0.000511)	(0.000496)	(0.000485)	(0.000478)
COPD	0.00575***	0.00700***	0.0105***	0.0142***	0.0183***	0.0239***
	(0.000284)	(0.000291)	(0.000294)	(0.000289)	(0.000289)	(0.000292)
SMI	-0.0616***	-0.0601***	-0.0599***	-0.0687***	-0.0729***	-0.0760***
	(0.000461)	(0.000490)	(0.000514)	(0.000527)	(0.000559)	(0.000595)
Diabetes	0.0233***	0.0239***	0.0257***	0.0267***	0.0281***	0.0303***
	(0.000245)	(0.000250)	(0.000252)	(0.000250)	(0.000251)	(0.000254)
Constant	0.251***	0.273***	0.275***	0.273***	0.291***	0.304***
	(0.000833)	(0.00469)	(0.00825)	(0.00746)	(0.00748)	(0.00759)
Observations	16,000,030	16,206,558	16,375,065	16,633,682	16,930,167	17,026,292
R-squared	0.039	0.036	0.036	0.037	0.036	0.036

Notes: Robust standard errors are displayed in parentheses below point estimates. County fixed effects are included in all regression models.
*** p<0.01, ** p<0.05, * p<0.1

© 2023 James HO et al. *JAMA Health Forum.*

eTable 2. Adjusted rate of disenrollment to TM by race/ethnicity and dual Medicaid enrollment

	Disenroll to TM	
	No ADRD	ADRD
Overall	4.0% (4.0 – 4.0)	5.0% (4.9 – 5.0)
Race/Ethnicity		
Non-Hispanic White	3.9% (3.9 - 3.9)	4.9% (4.8 - 4.9)
Black	4.5% (4.5 - 4.5)	5.6% (5.5 - 5.6)
Asian Pacific Islander	5.0% (4.9 - 5.1)	6.2% (6.1 - 6.2)
Hispanic	3.6% (3.6 - 3.7)	4.5% (4.5 - 4.5)
Other	4.6% (4.5 - 4.7)	5.7% (5.6 - 5.8)
Dual Status		
Non-Dual	3.0% (3.0 - 3.0)	3.7% (3.7 - 3.7)
Dual	6.3% (6.3 - 6.3)	7.8% (7.7 - 7.8)

Notes: Table shows the rate of disenrollment to TM by ADRD status stratified by race/ethnicity and dual status. Estimates were calculated using margins following the estimation of a pooled multinomial logistic regression with robust standard errors. 95% confidence intervals are shown below point estimates in parentheses.

© 2023 James HO et al. *JAMA Health Forum.*

eTable 3. Logistic regression estimating adjusted contract exit, 2013-2018

	2013 - 2014	2014 - 2015	2015 - 2016	2016 - 2017	2017 - 2018
Age					
Under 65			Ref.		
65-75	0.100	-0.421***	-0.0823	0.0429	-0.0768
	(0.131)	(0.145)	(0.156)	(0.140)	(0.131)
75-85	-0.122	-0.557***	-0.260*	-0.134	-0.257**
	(0.131)	(0.145)	(0.156)	(0.140)	(0.131)
85+	-0.341***	-0.690***	-0.446***	-0.315**	-0.469***
	(0.131)	(0.145)	(0.156)	(0.140)	(0.131)
Race/ethnicity					
White Non-Hispanic			Ref.		
Black	0.125***	0.0767***	0.123***	0.134***	0.185***
	(0.00481)	(0.00405)	(0.00446)	(0.00448)	(0.00453)
API	-0.171***	-0.0159*	0.119***	-0.0340***	0.0384***
	(0.0122)	(0.00933)	(0.00986)	(0.0105)	(0.0105)
Hispanic Non-White	0.154***	-0.0148***	0.263***	0.140***	0.284***
	(0.00504)	(0.00433)	(0.00457)	(0.00471)	(0.00472)
Other	-0.0334**	-0.0793***	-0.0234*	-0.0444***	0.0195
	(0.0149)	(0.0121)	(0.0132)	(0.0128)	(0.0127)
Female	-0.00720**	-0.00460*	-0.0143***	-0.0122***	-0.0371***
	(0.00325)	(0.00274)	(0.00313)	(0.00317)	(0.00328)
Dual enrollment	0.142***	0.200***	0.538***	0.362***	0.590***
	(0.00392)	(0.00324)	(0.00355)	(0.00364)	(0.00371)
Reason for entitlement					
OASI			Ref.		
DIB	0.205	-0.367**	-0.0101	0.127	0.0332
	(0.131)	(0.145)	(0.156)	(0.140)	(0.131)
ESRD	0.106	-0.443***	0.217	0.281**	0.259**
	(0.121)	(0.150)	(0.157)	(0.141)	(0.132)
Both DIB & ESRD	-0.338**	-0.710***	-0.613***	-0.318**	-0.617***
	(0.136)	(0.150)	(0.165)	(0.150)	(0.144)
ADRD	0.152***	0.144***	0.108***	0.123***	0.114***
	(0.00563)	(0.00463)	(0.00520)	(0.00516)	(0.00526)
Utilization					
Nursing home, Baseline	0.0242***	0.138***	0.107***	0.124***	0.129***
	(0.00520)	(0.00423)	(0.00480)	(0.00482)	(0.00502)

© 2023 James HO et al. *JAMA Health Forum.*

Nursing home, Y-1	-0.184***	-0.00450	-0.106***	-0.0862***	-0.101***
	(0.00587)	(0.00461)	(0.00539)	(0.00536)	(0.00560)
Inpatient, Baseline	-0.134***	-0.100***	-0.113***	-0.0895***	-0.0846***
	(0.00366)	(0.00307)	(0.00351)	(0.00356)	(0.00368)
Inpatient, Y-1	-0.113***	-0.0695***	-0.116***	-0.107***	-0.0862***
	(0.00360)	(0.00302)	(0.00347)	(0.00351)	(0.00363)
Home health, Baseline	-0.00402	-0.0684***	-0.0375***	-0.0432***	-0.0382***
	(0.00396)	(0.00333)	(0.00373)	(0.00379)	(0.00394)
Home health, Y-1	0.0435***	-0.0236***	0.0234***	0.0108***	0.00848**
	(0.00414)	(0.00348)	(0.00391)	(0.00397)	(0.00409)
Other health conditions					
Heart failure	-0.0105**	-0.0172***	-0.0135***	-0.00885**	-0.0185***
	(0.00445)	(0.00371)	(0.00419)	(0.00422)	(0.00431)
AMI	-0.0323***	-0.00645	-0.00776	-0.0111*	0.00413
	(0.00716)	(0.00592)	(0.00674)	(0.00665)	(0.00667)
COPD	0.0780***	0.0476***	0.0708***	0.0398***	0.0694***
	(0.00408)	(0.00345)	(0.00388)	(0.00388)	(0.00393)
SMI	0.0668***	0.101***	0.157***	0.148***	0.190***
	(0.00881)	(0.00729)	(0.00765)	(0.00800)	(0.00808)
Diabetes	0.0345***	0.0147***	0.0387***	0.0363***	0.0375***
	(0.00340)	(0.00286)	(0.00324)	(0.00329)	(0.00339)
Constant	-1.587***	-0.708***	-1.738***	-1.945***	-2.128***
	(0.131)	(0.145)	(0.156)	(0.140)	(0.131)
Observations	2,994,571	3,245,649	3,580,648	3,836,254	4,137,826

Notes: Robust standard errors are displayed in parentheses below point estimates which were log transformed to be interpreted as odds ratios.
*** p<0.01, ** p<0.05, * p<0.1

© 2023 James HO et al. *JAMA Health Forum.*

eTable 4. Adjusted rate of contract exit by race/ethnicity and dual Medicaid enrollment

	Contract Exit	
	No ADRD	ADRD
Overall	15.1%<br>(15.1 – 15.1)	16.3%<br>(16.2 – 16.3)
Race/Ethnicity		
Non-Hispanic White	14.6%<br>(14.5 – 14.6)	15.7%<br>(15.7 – 15.8)
Black	16.2%<br>(16.1 – 16.2)	17.5%<br>(17.4 – 17.5)
Asian Pacific Islander	14.7%<br>(14.6 – 14.9)	15.9%<br>(15.8 - 16.0)
Hispanic	16.8%<br>(16.8 – 16.9)	18.2%<br>(18.1 – 18.2)
Other	14.2%<br>(14.0 – 14.3)	15.3%<br>(15.2 – 15.5)
Dual Status		
Non-Dual	13.8%<br>(13.7 – 13.8)	14.9%<br>(14.8 – 14.9)
Dual	18.3%<br>(18.2 – 18.3)	19.7%<br>(19.6 – 19.7)

Notes: Table shows the rates of contract exit by ADRD status stratified by race/ethnicity and dual status. Estimates were calculated using margins following the estimation of a pooled logistic regression with robust standard errors. 95% confidence intervals are shown below point estimates in parentheses.

© 2023 James HO et al. *JAMA Health Forum.*

eMethods. Chronic Conditions Warehouse Criteria for Alzheimer's Disease and Related Disorders or Senile Dementia

(Source)

Reference Period:
3 years

Number/Type of Claims to Qualify:
At least 1 inpatient, SNF, HHA, HOP, or Carrier claim with DX code

Valid ICD-10 Codes:
F01.50, F01.51, F02.80, F02.81, F03.90, F03.91, F04, F05, F06.1, F06.8, G13.8, G30.0, G30.1, G30.8, G30.9, G31.01, G31.09, G31.1, G31.2, G94, R41.81, R54 (any DX on the claim)

Valid ICD-9 Codes:
331.0, 331.11, 331.19, 331.2, 331.7, 290.0, 290.10, 290.11, 290.12, 290.13, 290.20, 290.21, 290.3, 290.40, 290.41, 290.42, 290.43, 294.0, 294.10, 294.11, 294.20, 294.21, 294.8, 797 (any DX on the claim)

Acknowledgements

National Research Service Award from the Agency for Healthcare Research and Quality (Grant No. T32HS00011)

National Institute on Aging (Grant No. P01AG027296)

© 2023 James HO et al. *JAMA Health Forum.*
