# Supplementary material for: Medicare Advantage Enrollment and Disenrollment Among Persons With Alzheimer Disease and Related Dementias
Source: JAMA Health Forum. 2023 Sep 15;4(9):e233080. doi: 10.1001/jamahealthforum.2023.3080 (PMC10504614; doi:10.1001/jamahealthforum.2023.3080)
Supplement: Supplement 2. — Data Sharing Statement [file jamahealthforum-e233080-s002.pdf]

Data Sharing Statement

James. Medicare Advantage Enrollment and Disenrollment Among Persons with Alzheimer Disease and Related Dementias. *JAMA Health Forum*. Published September 15, 2023. doi:10.1001/jamahealthforum.2023.3080

Data
Data available: No

Additional Information
Explanation for why data not available: Consistent with our DUA, we are unfortunately not able to provide patient level data. Researchers interested in this data are able to apply for access to the data directly.
